# Outcomes after using cerebral embolic protection devices during transcatheter aortic valve replacement: an updated meta-analysis

**DOI:** 10.3389/fcvm.2026.1782285

**Published:** 2026-05-18

**Authors:** Mohamed Saad Sayed, Amro K. AlQurm, Waleed Mohaned Rasheed, Kenda Yousef Abedal- Kareem, Saeb M. Albsoul, Sara K. AlQurm, Shatha M. Ramadneh, Jude N. Haddad, Obadah Aqtash, Tulio Caldonazo

**Affiliations:** 1 Faculty of Medicine, Beni-Suef University, Beni Suef, Egypt; 2 School of Medicine, Jordan University of Science and Technology, Irbid, Jordan; 3 Faculty of Medicine, Jordan University of Science and Technology, Irbid, Jordan; 4 Faculty of Medicine, Yarmouk University, Irbid, Jordan; 5 Department of Cardiology, Baylor Scott & White, the Heart Hospital, Plano, TX, United States; 6 Department of Cardiothoracic Surgery, Jena University Hospital, Jena, Germany

**Keywords:** cerebral embolic protection, meta-analysis, randomized controlled trials, stroke, transcatheter aortic valve replacement

## Abstract

**Background:**

Cerebral embolic protection (CEP) devices have been developed to reduce periprocedural embolization through transcatheter aortic valve replacement (TAVR), yet their clinical benefit remains uncertain. This study aimed to systematically evaluate the efficacy and safety of CEP devices during TAVR using evidence restricted to randomized controlled trials (RCTs).

**Methods:**

We conducted a systematic review and meta-analysis following PRISMA guidelines. MEDLINE, Embase, Web of Science, Scopus, and Cochrane CENTRAL were searched through July 2025 for RCTs comparing CEP devices vs. no protection in patients undergoing TAVR. The primary outcome was the all-cause stroke. Random-effects model was applied for the primary analysis.

**Results:**

Nine RCTs comprising 11,696 patients (6,000 patients in CEP, 5,696 patients in control) were analyzed. CEP use did not significantly reduce the overall risk of all-cause stroke **(**RR 0.92; 95% CI 0.73–1.14; *p* = 0.43**).** The results were consistent across different subgroups, either Sentinel (filter device) **(**RR 0.88; 95% CI 0.70–1.11; I^2^ = 0.00%) or TriGuard (deflection device) **(**RR 1.40; 95% CI 0.67–2.94; I^2^ = 0.00%) (P_interaction_ = 0.50). Similarly, no significant differences between the two groups were observed for the risk of all-cause mortality, disabling stroke, non-disabling stroke, cardiovascular mortality, transient ischemic attack, major adverse cardiovascular and cerebrovascular events, major bleeding, major vascular complications, or acute kidney injury.

**Conclusions:**

Among patients undergoing TAVR, CEP devices could not reduce the risk of stroke compared with the control group.

**Systematic Review Registration:**

https://www.crd.york.ac.uk/PROSPERO/view/CRD420251114450, CRD420251114450.

## 1 Introduction

Aortic stenosis (AS) is a progressive disorder that leads to significant health complications and increased mortality (1). The underlying pathology mirrors atherosclerotic processes, characterized by lipid deposition, inflammation, and calcification (2). Transcatheter aortic valve replacement (TAVR) and surgical aortic valve replacement (SAVR) are well-established options for severe AS. According to the 2020 ACC/AHA guidelines, the choice between SAVR and TAVR should be individualized, incorporating patient-specific factors, procedural considerations, and prosthesis characteristics, and determined by a multidisciplinary heart team (3). In symptomatic patients aged 65–80 years without contraindications to transfemoral access, either SAVR or TAVR is recommended (Class I, LOE A) after shared decision-making about the balance between expected patient longevity and valve durability, while SAVR remains preferred for younger patients with longer life expectancy (3).

Although TAVR is generally safe, cerebrovascular events remain a major complication, leading to substantial increases in morbidity and a 3.5-fold higher mortality in the first postoperative month (4, 5). With the anticipated expansion of TAVR to younger and lower-risk patients, strategies for stroke prevention are becoming increasingly essential (6). Cerebral embolic protection devices (CEP devices) have been developed to minimize embolization during TAVR through two mechanisms: filtration systems, which capture and remove emboli, or deflection systems, which redirect debris away from cerebral circulation (7).

Current guideline recommendations for CEP devices remain inconsistent. The 2023 Saudi TAVR Guidelines (SHA, NHC, SACIS, SSCS, SCIG) suggest CEP devices use in selected high-risk populations, including patients with prior stroke/TIA, severe valvular calcification, or bicuspid valve anatomy, whereas most international guidelines have not issued formal recommendations (8). Earlier meta-analysis suggested stroke reduction without differences in disabling or non-disabling strokes (9). In contrast, the large PROTECTED-TAVR RCT, enrolling 7,635 patients, found no significant reduction in overall or disabling stroke with CEP devices use (10). These neutral findings conflict with earlier evidence suggesting stroke reduction (9, 11), adding to the uncertainty surrounding their clinical efficacy. Given these discrepancies, particularly with new high-powered randomized controlled trial (RCT) data, an updated meta-analysis restricted to RCTs is warranted to clarify the impact of CEP devices on outcomes in TAVR.

## 2 Methods

This systematic review was conducted according to the Preferred Reporting Items Systematic Reviews and Meta-Analyses (PRISMA) guidelines (12) and with adherence to the Cochrane handbook for systematic reviews (13). The protocol was prospectively registered in the PROSPERO international prospective register of systematic reviews ID (CRD420251114450). Ethical approval was waived because we analyzed the data from published studies.

### 2.1 Data sources and literature search

A comprehensive search of MEDLINE, Embase, Web of Science, Scopus, and Cochrane CENTRAL was conducted for studies published from inception till July 2025. Search terms combined the descriptors for TAVR and cerebral embolic protection devices: (“Transcatheter Aortic Valve Replacement” OR “TAVI” OR “TAVR”) AND (“Cerebral Protection Devices” OR “Sentinel Device”). The detailed search strategy is shown in Supplementary Table 1. Further manual search for the first 200 hits on Google Scholar using the search strategy was performed to retrieve any missed trials that would be eligible for inclusion. Also, the screening of the references and citations of included studies and previous meta-analyses was conducted by two independent reviewers to identify any dropped studies from the search and selection process.

### 2.2 Eligibility criteria and study selection

Studies that were eligible for inclusion were RCTs that studied patients who underwent TAVR, involved the use of CEP devices compared to the non-CEP devices (control). There were no restrictions on valve type or access routes. Only full-text English-language articles were included. Studies were excluded if they were single-arm studies, observational studies (case reports/series, case control, or cohort studies), any systematic or literature reviews, or editorials without primary data.

All retrieved records were imported into Rayyan.ai (14) for screening. Two investigators (J.H. and S.R.) independently and blindly reviewed titles and abstracts for relevance. Full-text articles were obtained for all studies that met the inclusion criteria or when eligibility remained uncertain. Disagreements were resolved through discussion and, if necessary, solved by a third reviewer (A.Q).

### 2.3 Data extraction and outcomes

Data was independently extracted by two reviewers using a standardized template. Extracted information included study characteristics, patient demographics, and outcomes of interest. The primary outcome was all-cause stroke. that is defined according to the criteria established by the Valve Academic Research Consortium 2 (VARC 2) (15) and the Neurologic Academic Research Consortium (NeuroARC) (16), was reported at the most extended duration of follow-up. Other clinical outcomes were all-cause mortality, disabling stroke, non-disabling stroke, hemorrhagic stroke, ischemic stroke, cardiovascular mortality, transient ischemic attack (TIA), major adverse cardiovascular and cerebrovascular events (MACCE), major bleeding, major vascular complications, or acute kidney injury (AKI).

### 2.4 Quality assessment and certainty of evidence

The risk of bias of the included studies was assessed using the Cochrane ROB-2 tool, which evaluates potential bias across domains such as randomization, deviations from intended interventions, missing data, outcome measurement, and selective reporting (17). The certainty of the body of evidence was subsequently appraised using the GRADE approach in GRADEpro, which considers study limitations, consistency of results, directness, precision, and publication bias (18).

### 2.5 Statistical analysis

The event and total of the dichotomous data were pooled using risk ratios (RRs), with corresponding 95% confidence intervals (CIs) were calculated for each outcome of interest. A random-effects model [restricted maximum likelihood (REML)] was primarily used to account for potential heterogeneity among the included studies. Secondary analyses were conducted using a fixed-effects model (Mantel-Haenszel Model) to test the robustness of the results. The statistical heterogeneity was assessed using the I^2^ statistic, as well as the Chi-square test, with values of 25%, 50%, and 75% representing low, moderate, and high heterogeneity, respectively (19). A leave-one-out sensitivity analysis was performed to evaluate the influence of individual studies on the overall estimates. Subgroup analyses were conducted based on the specific types of devices used [e.g., Sentinel (filter device) vs. TriGuard (deflection device)], and follow-up duration (in hospital vs. within 30-day vs. within 90-day after TAVR) for the primary outcome (all-cause stroke). Random effects meta-regression were performed to show the association between the incidence of all-cause stroke and study covariates, including mean age, male sex, diabetes, hypertension, atrial fibrillation or flutter, prior PCI, prior CABG, prior stroke or TIA, EuroSCORE II, and STS score. Funnel plots were generated for key outcomes to assess publication bias. In case of suspicion of publication bias, the trim-and-fill method was used to estimate the potential impact of missing studies (20). The numerical publication bias was assessed using Egger's regression test (21) and Begg's Rank correlation tests (22). A *p*-value < 0.05 was considered statistically significant for all analyses. All analyses were conducted using STATA Corp M17 (23).

## 3 Results

### 3.1 Search results

**Figure 1:**
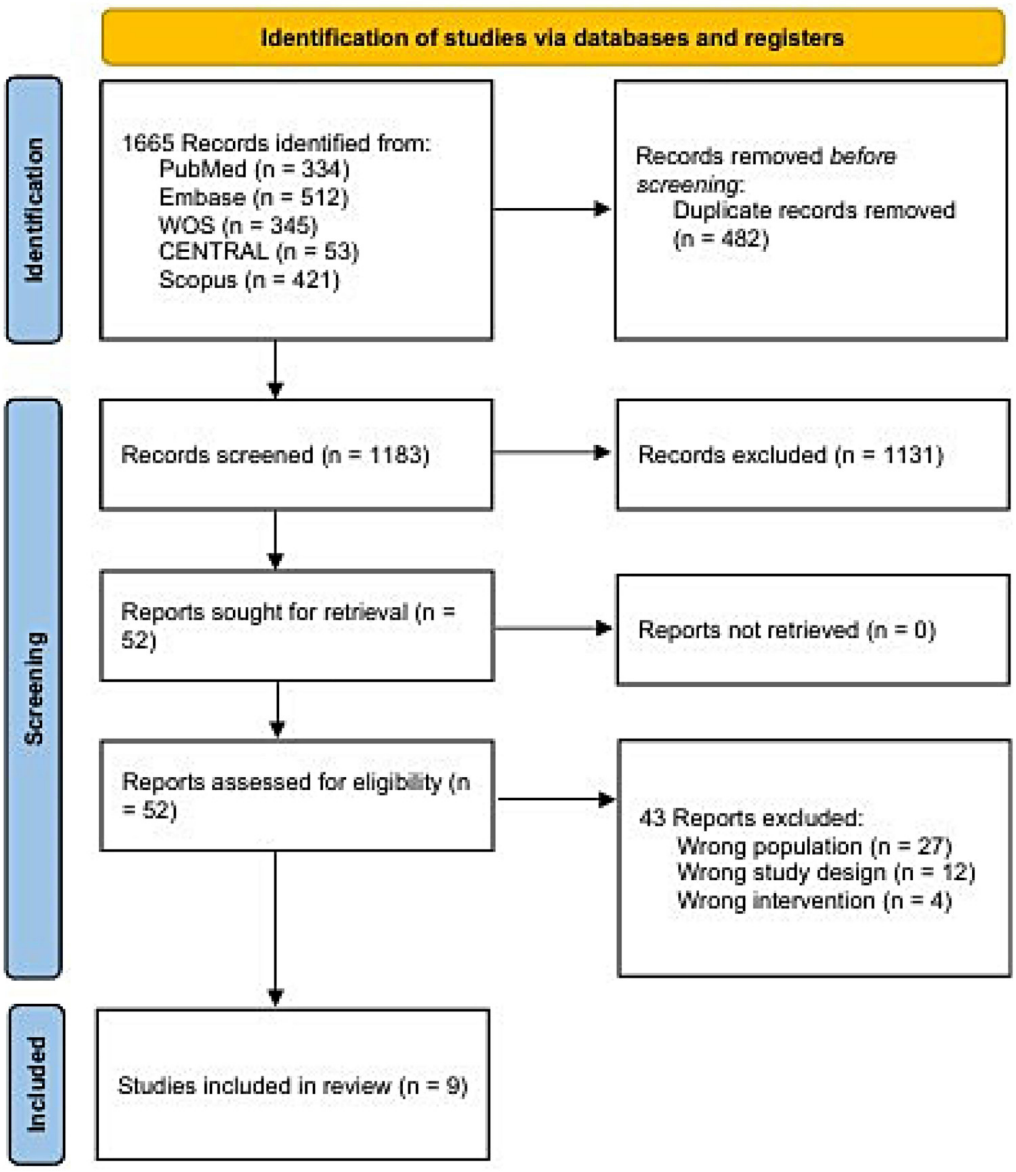
PRISMA flowchart for selection process.

From 1,665 initial records, 482 duplicates were removed using Rayyan. After screening, 52 studies were assessed in full text. The primary reasons for exclusion at this stage were wrong population and inappropriate study design (non-RCTs, reviews, retrospective or prospective studies). Of the 52 studies assessed, 9 RCTs (10, 24–31) met the eligibility criteria and were included in the final analysis. The study selection process is summarized in the PRISMA flow diagram (Figure 1).

### 3.2 Study characteristics

A total of 9 included trials (10, 24–31) comprising a total of 11,696 patients were included in this systematic review and meta-analysis, with 6,000 patients receiving CEP devices and 5,696 in the control group. The patients’ mean age was 81.20 (6.65) years, while the males were 59.7% of the whole patients. The follow-up ranged from 30 to 90 days. A total of 2 trials (24, 25) were single-center trials conducted in Germany, while the remaining 7 trials were multicenter studies (10, 26–30, 32) involving sites across the United Kingdom, Germany, the Netherlands, Europe, Australia, and Canada. The detailed characteristics of these studies are provided in Tables 1, 2.

**Table 1:** Summary of included studies.

Study ID	Trial name	Study design	Country	Groups (n)			Device type	TAVR route	Stroke definition
				Total	CEP devices	Control group			
Kharbanda et al. 2025 (10)	BHF PROTECT-TAVI	RCT	United Kingdom	7,635	3,815	3,820	Sentinel Cerebral Protection System (CPS)	Transfemoral	VARC-2
Kapadia et al. 2022 (29)	PROTECTED TAVR	RCT	North America, Europe, and Australia	3,000	1,501	1,499	Sentinel Cerebral Protection System (CPS)	Transfemoral	NeuroARC
Nazif et al. 2021 (26)	REFLECT II	RCT	USA	220	162	58	TriGuard HDH	Transfemoral	VARC-2
Lansky et al. 2021 (27)	REFLECT I	RCT	USA and Europe	258	141	63	TriGuard HDH	Transfemoral	VARC-2 NeuroARC
Kapadia et al. 2017 (31)	SENTINEL	RCT	USA and Germany	363	244	119	Claret Sentinel	Transfemoral	VARC-2
Haussig et al. 2016 (24)	CLEAN-TAVI	RCT	Germany	100	50	50	Claret Montage	Transfemoral	VARC-2
Meighem et al. 2016 (30)	MISTRAL-C	RCT	Netherlands	65	32	33	Sentinel Cerebral Protection System (CPS)	Transfemoral	VARC-2
Lansky et al. 2015 (28)	DEFLECT III	RCT	Europe and Israel	85	46	39	TriGuard HDH	Transfemoral	VARC-2
Wendt et al. 2015 (25)	EMBOL-X	RCT	Germany	30	14	16	EMBOL-X	Transaortic	VARC-2

VARC 2, Valve Academic Research Consortium 2; NeuroARC, the Neurologic Academic Research Consortium; RCT, randomized controlled trial.

**Table 2:** Baseline characteristics of patients included in the studies.

CEP devices/Control																
Study ID	Trial Name	Patient Demographics				Risk Scores			Comorbidities							
		Sample Size	Mean Age (SD)	Female no. (%)	Mean BMI (SD)	STS PROM score %	Logistic Euro SCORE-I,%	Euro SCORE-II,%	Atrial Fibrillation no. (%)	Hypertension no. (%)	DM no. (%)	CAD no. (%)	Previous stroke (cerebrovascular stroke) no. (%)	PVD no. (%)	Previous coronary revascularization, no. (%)	mean LVEF (SD)
Kharbanda et al. 2025 (10)	BHF PROTECT - TAVI	7,601 (3,798/3,803)	81.2 ± 6.5/81.3 ± 6.5	1,484 (39.1%)/1,461 (38.4%)	-	-	-	median: 2.4 (1.6–4.1)/2.4 (1.6–4.0)	1,256 (33.5%)/1,269 (33.8%)	2,558 (68.4%)/2,528 (67.4%)	793 (20.9%)/767 (20.2%)	1,234 (34.6%)/1,168 (32.9%)	217 (5.8%)/235 (6.3%)	262 (7.7%)/255 (7.5%)	-	-
Kapadia et al. 2022 (29)	PROTECTED TAVR	3,000 (1,501/1,499)	78.9 ± 8/78.9 ± 7.8	631 (42%)/566 (37.8%)	-	3.3 ± 2.7/3.4 ± 2.8	-	-	511 (34.1%)/469 (31.4%)	1,306 (87.1%)/1,312 (87.6%)	501 (33.4%)/522 (34.8%)	850 (56.9%)/880 (58.9%)	114 (7.6%)/122 (8.2%)	165 (11.1%)/162 (10.9%)	495 (33.1%)/548 (36.6%)	-
Nazif et al. 2021 (26)	REFLECT II	214 (157/57)	80.31 ± 7.73/78 ± 8.19	71 (45.2%)/22 (38.6%)	-	4.64 ± 2.77/4.54 ± 2.50	-	3.76 ± 3.08/3.59 ± 3.41	44 (28%)/17 (29.8%)	-	61 (39.1%)/23 (40.4%)	30 (19.9%)/13 (23.2%)	17 (10.8%)/2 (3.5%)	20 (12.9%)/11 (19.3%)	-	-
Lansky et al. 2021 (27)	REFLECT I	204 (141/63) [excluding roll in]	79.8 ± 7.3/81.5 ± 7.1	61 (43.3%)/21 (33.3%)	-	4.6 ± 2.8/4.8 ± 3.1	-	4.8 ± 4.1/5.5 ± 4.1	45 (33.1%)/16 (25.8%)	-	60 (42.9%)/20 (31.7%)	-	10 (7.5%)/4 (6.7%)	15 (11.2%)/8 (13.6%)	-	57.8 ± 10.8/56.2 ± 11.8
Kapadia et al. 2017 (31)	SENTINEL	363 (244/119)	82.37 ± 7.93/84.23 ± 8.25	53.7/ 48.7	27.0 ± 5.96/27.1 ± 5.02	5.86 ± 3.03/6.57 ± 3.07	-	-	79 (32.38)/36 (30.3)	-	40 (16.39)/45 (37.8)	127 (52.05)/66 (55.5)	15 (6.15)/6 (5)	37 (15.16)/18 (15.1)	80 (32.79)/45 (37.82)	-
Haussig et al. 2016 (24)	CLEAN-TAVI	100 (50/50)	80 ± 5.1/79.3 ± 4.1	29 (58)/28 (56)	-	5.6 ± 3.2/5.2 ± 2.7	16.4% ± 10.0/ 14.5% ± 8.7	-	(A-fib or Atrial flutter) 17 (34)/17 (34)	44 (88)/47 (94)	20 (40)/25 (50)	26 (52)/25 (50)	1 (2)/3 (6)	2 (4)/4 (8)	13 (26)/ 10 (20)	-
Meighem 2016 (30)	MISTRAL-C	65 (32/33)	81.67 ± 3.88/81.9 ± 3.88	15 (47)/16 (49)	-	4.77 ± 2.25/6.37 ± 4.88	-	-	8 (29)/8 (27)	21 (66)/23 (70)	4 (13)/9 (27)	-	6 (19)/6 (18)	9 (28)/11 (33)	-	57 ± 14/53 ± 16
Lansky et al. 2015 (28)	DEFLECT III	85 (46/39)	82.5 ± 6.5/82.3 ± 6	26 (56.5%)/20 (51.3%)	-	6.3 ± 5.8/7.4 ± 5.5	-	10.1 ± 10.1/7.2 ± 6.6	9.98 (21.7%)/14 (35.9%)	36.9 (80.4%)/28 (71.8%)	9.98 (21.7%)/9 (23.1%)	-	6.118 (13.3%)/6.98 (17.9%)	5.98 (13%)/4.99 (12.8%)	-	-
Wendt et al. 2015 (25)	EMBOL-X	30 (14/16)	81.0 ± 5.0/82.1 ± 4.1	10 (71.4)/ 8 (50.0)	-	11.4 ± 6.9/9.3 ± 6.3	39.2 ± 13.0/39.5 ± 10.5	8.6 ± 3.9/8.1 ± 5.6	-	-	-	-	1 (7.1)/3 (12.5)	5 (35.7)/6 (37.5)	-	0.498 ± 0.121/0.531 ± 0.080

### 3.3 Quality assessment and certainty of evidence

**Figure 2:**
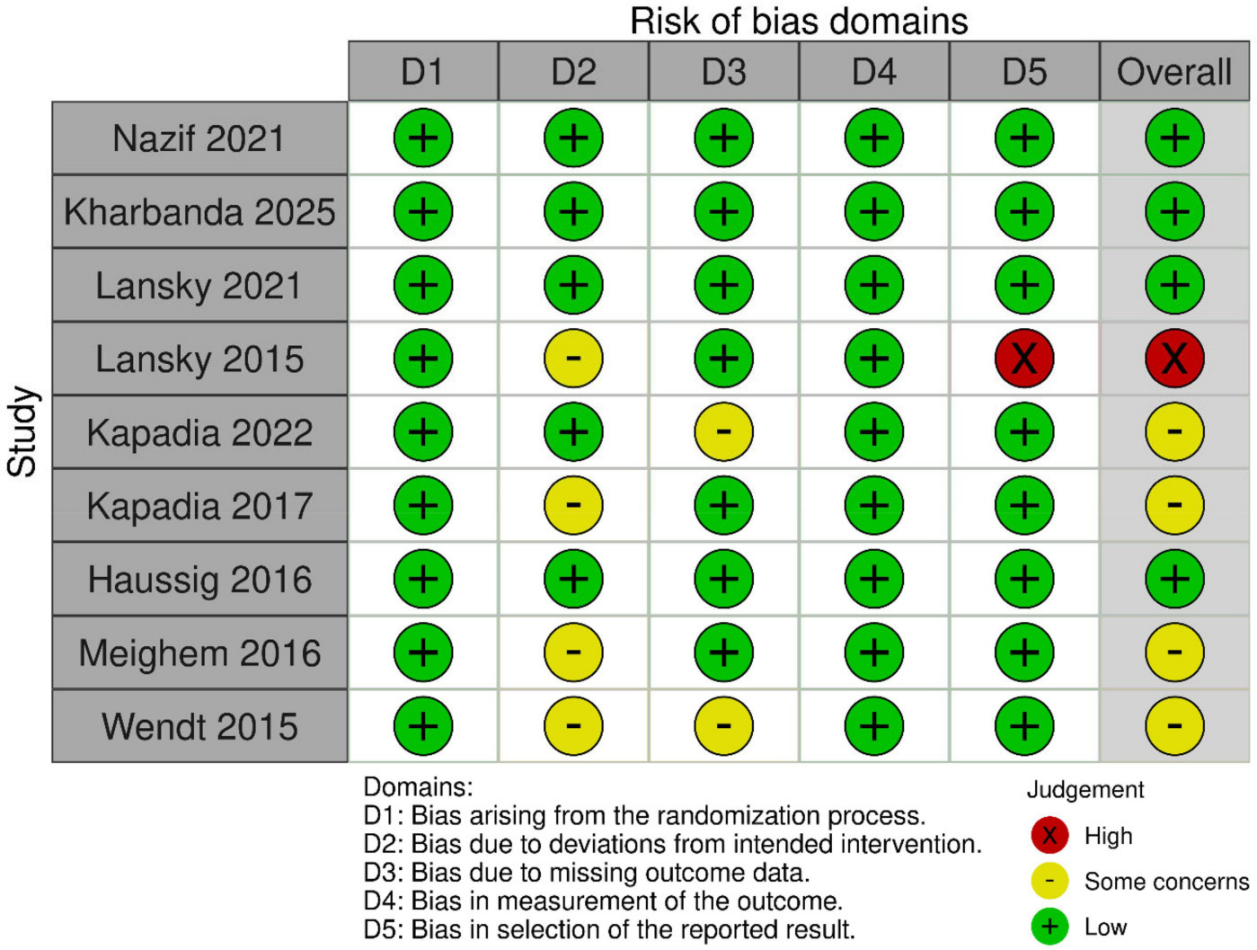
Risk of bias assessment of included studies.

The quality of the included studies was assessed using the Cochrane ROB-2 quality assessment tool. The risk of bias for the four studies (10, 24, 26, 27) were determined to be “low risk”, and the other four studies (25, 29–31) had “some concerns” at overall risk of bias with one study (28) assessed as “high risk” (Figure 2). We applied the GRADE approach in GRADEpro to evaluate the evidence quality. All the assessed outcomes were of high-quality evidence, except that TIAs were of moderate certainty. Supplementary Table 2 shows the GRADE assessment for the analyzed outcomes.

### 3.4 Outcomes

#### 3.4.1 Primary outcome

##### 3.4.1.1 All-Cause stroke

**Figure 3:**
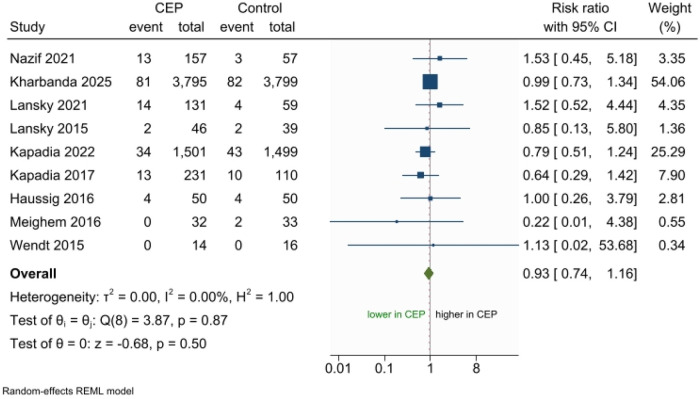
Forest plot meta-analysis for incidence of stroke.

All studies reported the risk of all-cause stroke. The pooled analysis using a random-effects model showed no significant difference between the CEP devices and control groups regarding the risk of all-cause stroke **(**RR 0.92; 95% CI 0.73–1.14; *p* = 0.43**),** with no observed heterogeneity among the studies **(**I^2^ = 0%) (Figure 3). The results were consistent across the leave-one-out analysis, which revealed that excluding any study did not affect the direction of the overall result (Supplementary Figure 1). Secondary analysis using the fixed-effects model also yielded consistent results (Supplementary Figure 2).

Subgroup analyses were performed based on specific types of devices and follow-up duration. Regarding the types of devices, there was no significant difference regarding the incidence of all-cause stroke between CEP devices and control group either when using Sentinel (filter device) **(**RR 0.88; 95% CI 0.70–1.11; I^2^ = 0.00%) or TriGuard (deflection device) **(**RR 1.40; 95% CI 0.67–2.94; I^2^ = 0.00%) (P_interaction_ = 0.50) (Supplementary Figure 3). Similarly, there was no statistical difference between CEP devices and control group regarding the incidence of all-cause stroke across different follow-up durations: in hospital (RR 0.95; 95% CI 0.75–1.20; I^2^ = 0.00%) vs. within 30-day (RR 0.89; 95% CI 0.55–1.43; I^2^ = 0.00%) vs. within 90-day after TAVR (RR 1.62; 95% CI 0.56–4.68; I^2^ = 0.00%) (P_interaction_ = 0.60) (Supplementary Figure 4).

The visual depiction of the funnel plot revealed symmetrical distribution, and without any imputed studies after trim and fill analysis (Supplementary Figure 5). In addition, there was no significance in Egger's regression test (*p*-value = 0.89) and Begg's correlation test (*p*-value = 0.60), which revealed that no numerical publication bias was observed (Supplementary Table 3). Random effects meta-regression revealed that no significant association between the incidence of all-cause stroke and study covariates, including mean age (*p* = 0.65), male sex (*p* = 0.78), diabetes (*p* = 0.46), hypertension (*p* = 0.78), atrial fibrillation or flutter (*p* = 0.69), prior PCI (*p* = 0.54), prior CABG (*p* = 0.66), prior stroke or TIA (*p* = 0.91), EuroSCORE II (*p* = 0.43), and STS score (*p* = 0.51).

#### 3.4.2 Secondary outcomes

**Figure 4:**
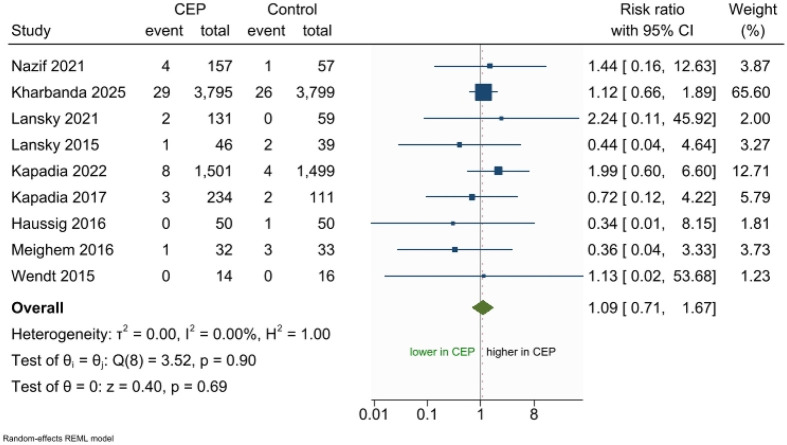
Forest plot meta-analysis for incidence of All-cause mortality.

**Figure 5:**
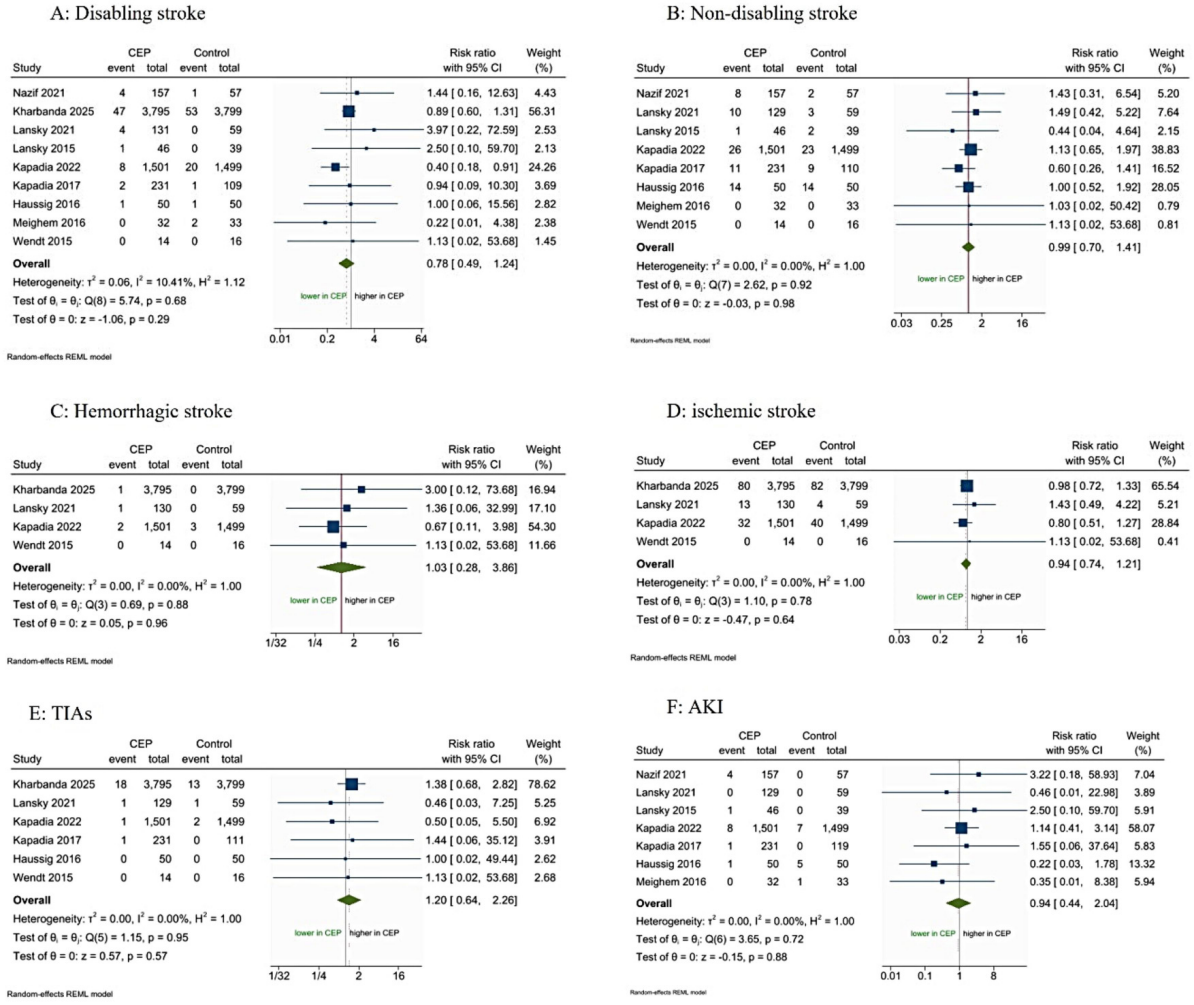
Forest plot meta-analysis for incidence of **(A)** disabling stroke, **(B)** non-disabling stroke, **(C)** hemorrhagic stroke, **(D)** ischemic stroke, **(E)** transient ischemic strokes (TIAs), and **(F)** acute kidney injury (AKI).

There were no statistical differences between the CEP devices and control group in the incidence of all-cause mortality (RR 1.09; 95% CI 0.71–1.67; *p* = 0.69, I^2^ = 0%, Figure 4), disabling stroke (RR 0.78; 95% CI 0.49–1.24; *p* = 0.29, I^2^ = 10.41%, Figure 5A), nondisabling stroke (RR 0.99; 95% CI 0.70–1.41; *p* = 0.98, I^2^ = 0.00%, Figure 5B), hemorrhagic stroke (RR 1.03; 95% CI 0.28–3.86; *p* = 0.96; I^2^ = 0.00%, Figure 5C), ischemic stroke (RR 0.94; 95% CI 0.74–1.21; *p* = 0.64; I^2^ = 0.00%, Figure 5D), TIAs (RR 1.20; 95% CI 0.64–2.26; *p* = 0.57, I^2^ = 0.00%, Figure 5E), AKI (RR 0.94; 95% CI 0.44–2.04; *p* = 0.88; I^2^ = 0.00%, Figure 5F), cardiovascular mortality (RR 1.58; 95% CI 0.59–4.20; *p* = 0.36, I^2^ = 0.00%, Supplementary Figure 6A), MACCE (RR 1.19; 95% CI 0.78–1.81; *p* = 0.43, I^2^ = 68.28%, Supplementary Figure 6B), major bleeding (RR 0.97; 95% CI 0.47–1.97; *p* = 0.92, I^2^ = 0.00%, Supplementary Figure 6C), and major vascular complications (RR 1.17; 95% CI 0.78–1.75; *p* = 0.44, I^2^ = 0.00%, Supplementary Figure 6D). The results were consistent in the secondary analysis using the fixed-effects model (Supplementary Table 4).

## 4 Discussion

This systematic review and meta-analysis relied on RCTs that compared the CEP devices with control groups in patients undergoing TAVR to evaluate the clinical outcomes after TAVR, which included 9 RCTs encompassing 11,696 patients (6,000 patients in CEP devices, and 5,696 patients in control). The CEP devices were associated with a similar risk for all-cause stroke compared with the control group. The results were consistent across sensitivity analyses and subgroup analyses, regardless of the device used and follow-up duration. Furthermore, the incidence of other outcomes was similar between the two groups, such as all-cause mortality, disabling, non-disabling, hemorrhagic, and ischemic stroke risk. Regarding the safety outcomes, there were no differences between the two groups in major bleeding, major vascular complications, cardiovascular mortality, and AKI.

Stroke is a feared complication following TAVR, with randomized trial–based estimates of periprocedural stroke ranging between approximately 2% and 6%, depending on patient risk and trial design (32, 33). These events have serious clinical implications, such as the stroke after TAVR is associated with more than a threefold increase in short-term mortality, which is reported as 25.5% vs. 6.9% for those without stroke, longer hospital and ICU stays, and enduring functional and cognitive impairments (5, 34, 35). Previous meta-analyses that included observational cohorts had suggested potential benefits of CEP devices to reduce the risk of stroke in patients undergoing TAVR. Basit et al., showed that CEP devices during TAVR were associated with a significant reduction in the risk of stroke compared with the control group through analysis of 17 studies (7 RCTs and 10 cohort studies, with 155,829 patients) (9). Similarly, Zahid et al., showed a higher benefit of CEP devices usage through TAVR through a significant reduction in the risk of stroke, major adverse cardiovascular events, and all-cause mortality (11).

The most recent trial, the BHF PROTECT-TAVI trial, investigated the use of CEP devices for patients undergoing TAVR, using 29.6% of patients undergoing TAVR at participating NHS centers. The analysis included 7,635 patients across 33 UK centers, which gives the trial the high reproducibility of the results. Aligning with our results, the risk of stroke was similar between CEP devices and control groups across the primary analysis. Furthermore, other clinical outcomes such as all-cause mortality and disabling stroke were similar between the two groups (10).

The pooled analysis demonstrated no statistically significant reduction in overall stroke incidence, all-cause mortality, disabling or nondisabling stroke, TIAs, cardiovascular mortality, or MACCE with the use of CEP devices. It was against the previous meta-analyses that showed the higher efficacy of CEP devices in reducing the risk of strokes and all-cause mortality. The observed differences were elicited by including observational studies in past pooled analyses. Notably, the observed reduction in incidence of the stroke and mortality appeared largely driven by observational studies rather than randomized trials. The analysis conducted by Caminiti et al., showed similar results of our analysis (36). Regarding the safety outcomes, CEP devices have no significant differences compared to the control group through TAVR. According to the type of the device, subgroup analysis revealed consistent results through a non-significant interaction between Sentinel and TriGuard. The benefit of CEP devices to reduce the stroke has been developed by capturing embolic debris or deflecting it away from cerebral circulation. According to this analysis, there were no differences between two systems according to the risk of stroke. Moreover, there are a few reasons that could explain why CEP didn't lead to better clinical outcomes in our review. One important factor is that most devices, like the Sentinel, only protect two of the three main cerebral arteries, leaving the vertebrobasilar system exposed (37, 38). This means embolic debris can still reach the brain even when a device is used. Also, device placement isn't always successful; anatomical issues like difficult angles or vessel abnormalities prevented full deployment in some patients (31). To be addressed, the aortic valve area is not the only source of emboli, left atrium, or even during wire or catheter manipulation in other parts of the circulation, might be the source of emboli.

### 4.1 Strengths and limitations

While this review is vital in assessing the efficacy of CEP devices in TAVR, several limitations should be considered when interpreting these findings. First, several studies had relatively small sample sizes. Second, the short follow-up period ranged from 72 h to 30 days after TAVR. Third, two studies were single-center trials. Fourth, the variability in procedural techniques and operator experience may elicit difficulties for the patients who are suitable for CEP devices. Fifth, the studies were conducted in Europe and North America, while the data is limited to Africa and Asia, which limits the generalizability and applicability of the results of this analysis. Sixth, there was a clinical heterogeneity between the studies regarding the population characteristics, type of CEP devices, and follow-up duration; the statistical heterogeneity was absent or low.

### 4.2 Future implications and recommendations

According to this analysis, clinicians should use CEP devices with caution and under individualized approaches, considering the cost-benefit ratio, based on the patients’ clinical status and the risk of clinical complications after the procedure.

## 5 Conclusion

Among patients undergoing TAVR, CEP devices could not reduce the risk of stroke compared with the control group. Selective use in high-risk patients may still be beneficial; however, the need for larger multi-center trials with stratified subgroup analyses could improve the evidence around CEP devices usage.

## Data Availability

The original contributions presented in the study are included in the article/Supplementary Material, further inquiries can be directed to the corresponding author.
