# Supplementary material for: Outcomes after using cerebral embolic protection devices during transcatheter aortic valve replacement: an updated meta-analysis
Source: Front Cardiovasc Med. 2026 May 18;13:1782285. doi: 10.3389/fcvm.2026.1782285 (PMC13223537; doi:10.3389/fcvm.2026.1782285)

**SUPPLEMENTARY MATERIAL**

**Supplemental Table 1:** Search strategy for each database.

| Database | Search strategy | Fields | Records |
| --- | --- | --- | --- |
| PubMed | ("Transcatheter Aortic Valve Replacement" OR "Transcatheter Aortic Valve Implantation" OR "TAVI" OR "TAVR") AND ("Cerebral Protection Devices" OR "Embolic Protection Devices" OR "Cerebral Embolic Protection" OR "Claret Device" OR "TriGuard" OR "Sentinel Device") | ALL Fields | 334 |
| Embase | ("Transcatheter Aortic Valve Replacement" OR "Transcatheter Aortic Valve Implantation" OR "TAVI" OR "TAVR") AND ("Cerebral Protection Devices" OR "Embolic Protection Devices" OR "Cerebral Embolic Protection" OR "Claret Device" OR "TriGuard" OR "Sentinel Device") | Title, abstract, and keywords | 512 |
| WOS | ("Transcatheter Aortic Valve Replacement" OR "Transcatheter Aortic Valve Implantation" OR "TAVI" OR "TAVR") AND ("Cerebral Protection Devices" OR "Embolic Protection Devices" OR "Cerebral Embolic Protection" OR "Claret Device" OR "TriGuard" OR "Sentinel Device") | ALL Fields | 345 |
| Scopus | ("Transcatheter Aortic Valve Replacement" OR "Transcatheter Aortic Valve Implantation" OR "TAVI" OR "TAVR") AND ("Cerebral Protection Devices" OR "Embolic Protection Devices" OR "Cerebral Embolic Protection" OR "Claret Device" OR "TriGuard" OR "Sentinel Device") | Title, abstract, and keywords | 421 |
| Cochrane Central | ("Transcatheter Aortic Valve Replacement" OR "Transcatheter Aortic Valve Implantation" OR "TAVI" OR "TAVR") AND ("Cerebral Protection Devices" OR "Embolic Protection Devices" OR "Cerebral Embolic Protection" OR "Claret Device" OR "TriGuard" OR "Sentinel Device") | Title, abstract, and keywords | 53 |

**Supplemental Table 2:** GRADE assessment in a separate file.

| **Certainty assessment** | | | | | | | **№ of patients** | | **Effect** | | **Certainty** | **Importance** |
| --- | --- | --- | --- | --- | --- | --- | --- | --- | --- | --- | --- | --- |
| **№ of studies** | **Study design** | **Risk of bias** | **Inconsistency** | **Indirectness** | **Imprecision** | **Other considerations** | **CEP** | **Control** | **Relative (95% CI)** | **Absolute (95% CI)** |  |  |
| **Stroke** | | | | | | | | | | | | |
| 9 | randomised trials | not serious | not serious | not serious | not serious | none | 163/5957 (2.7%) | 156/5662 (2.8%) | **RR 0.92** (0.73 to 1.14) | **2 fewer per 1,000** (from 7 fewer to 4 more) | ⨁⨁⨁⨁ High | CRITICAL |
| **All-cause mortality** | | | | | | | | | | | | |
| 9 | randomised trials | not serious | not serious | not serious | not serious | none | 48/5960 (0.8%) | 39/5663 (0.7%) | **RR 1.09** (0.71 to 1.67) | **1 more per 1,000** (from 2 fewer to 5 more) | ⨁⨁⨁⨁ High | CRITICAL |
| **MACCE** | | | | | | | | | | | | |
| 6 | randomised trials | not serious | not serious | not serious | not serious | none | 176/652 (27.0%) | 109/349 (31.2%) | **RR 1.19** (0.78 to 1.81) | **59 more per 1,000** (from 69 fewer to 253 more) | ⨁⨁⨁⨁ High | CRITICAL |
| **TIAs** | | | | | | | | | | | | |
| 6 | randomised trials | not serious | not serious | not serious | serious^a^ | none | 21/5720 (0.4%) | 16/5534 (0.3%) | **RR 1.20** (0.64 to 2.26) | **1 more per 1,000** (from 1 fewer to 4 more) | ⨁⨁⨁◯ Moderate^a^ | CRITICAL |
| **Disabling stroke** | | | | | | | | | | | | |
| 9 | randomised trials | not serious | not serious | not serious | not serious | none | 67/5957 (1.1%) | 78/5661 (1.4%) | **RR 0.78** (0.49 to 1.24) | **3 fewer per 1,000** (from 7 fewer to 3 more) | ⨁⨁⨁⨁ High | CRITICAL |
| **Nondisabling stroke** | | | | | | | | | | | | |
| 8 | randomised trials | not serious | not serious | not serious | not serious | none | 70/2160 (3.2%) | 53/1863 (2.8%) | **RR 0.99** (0.70 to 1.41) | **0 fewer per 1,000** (from 9 fewer to 12 more) | ⨁⨁⨁⨁ High | CRITICAL |

**CI:** confidence interval; **RR:** risk ratio

#### Explanations

a. Number of events is relatively low in most studies.

| **Outcome** | **P-values** | |
| --- | --- | --- |
|  | **Egger’s regression test** | **Beg’s rank correlation test** |
| Stroke | 0.89 | 0.60 |
| Disabling stroke | 0.53 | 0.91 |
| Non-Disabling stroke | 0.89 | 0.53 |
| All-cause mortality | 0.56 | 0.60 |
| MACCE | 0.12 | 0.25 |
| AKI | 0.86 | 0.99 |
| Major vascular complications | 0.11 | 0.22 |
| Major bleeding | 0.21 | 0.45 |
| CVS mortality | 0.55 | 0.22 |
| TIAs | 0.54 | 0.99 |
| Ischemic stroke | 0.76 | 0.99 |
| Hemorrhagic stroke | 0.59 | 0.73 |

**Supplemental Table 3:** Tests for the Small study effects

**Supplemental Table 4:** Secondary analysis using the fixed effects model.

| **Outcome** | **RR; 95% CI** | **P-value** | **I^2^** |
| --- | --- | --- | --- |
| All-cause mortality | 1.09; [0.72; 1.66] | 0.69 | 0% |
| Disabling stroke | 0.80 [0.58; 1.11] | 0.18 | 0% |
| Non-Disabling stroke | 1.00; [0.71; 1.42] | 0.89 | 0% |
| MACCE | 1.16; [0.93; 1.44] | 0.18 | 64.61% |
| AKI | 0.92; [0.45; 1.88] | 0.82 | 0% |
| Major vascular complications | 1.40; [0.94; 2.08] | 0.1 | 14.06% |
| Major bleeding | 1.15; [0.59; 2.23] | 0.68 | 4.10% |
| CVS mortality | 1.55; [0.60; 3.99] | 0.36 | 0% |
| TIAs | 1.20; [0.64; 2.24] | 0.56 | 0% |
| Ischemic stroke | 0.94; [0.74; 1.21] | 0.64 | 0% |
| Hemorrhagic stroke | 1.07; [0.30; 3.79] | 0.92 | 0% |

**Supplemental Figure 1:** Sensitivity analysis using leave-one-out methodology for All-cause stroke.


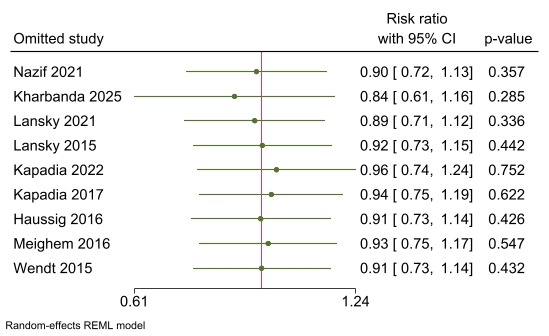


**Supplemental Figure 2:** Secondary analysis using the fixed effects model for All-cause stroke.


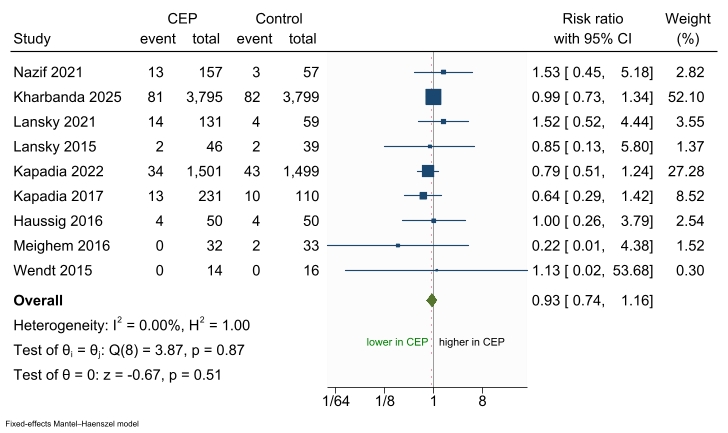


**Supplemental Figure 3:** Subgroup analysis based on type of device for all-cause stroke.


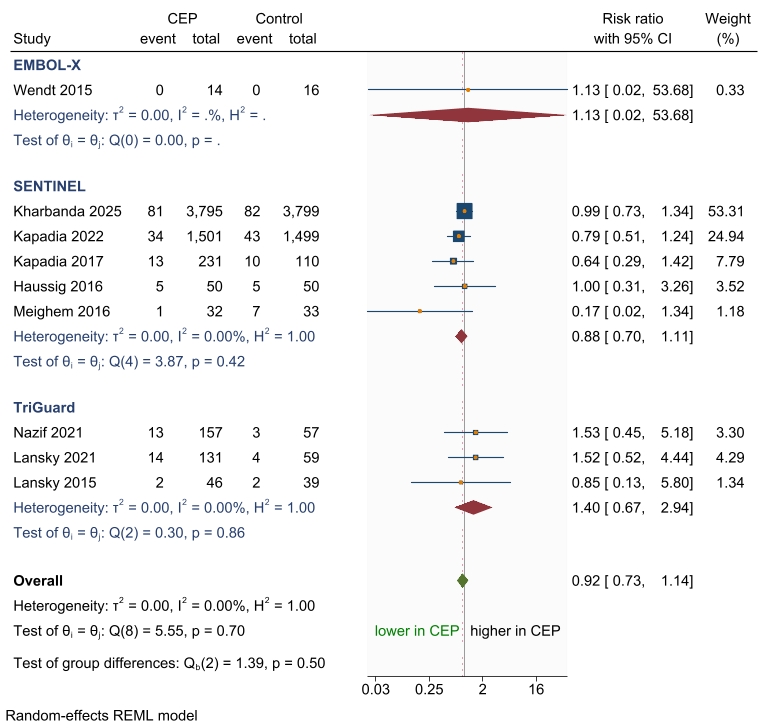


**Supplemental Figure 4:** Subgroup analysis based on follow-up duration for all-cause stroke.


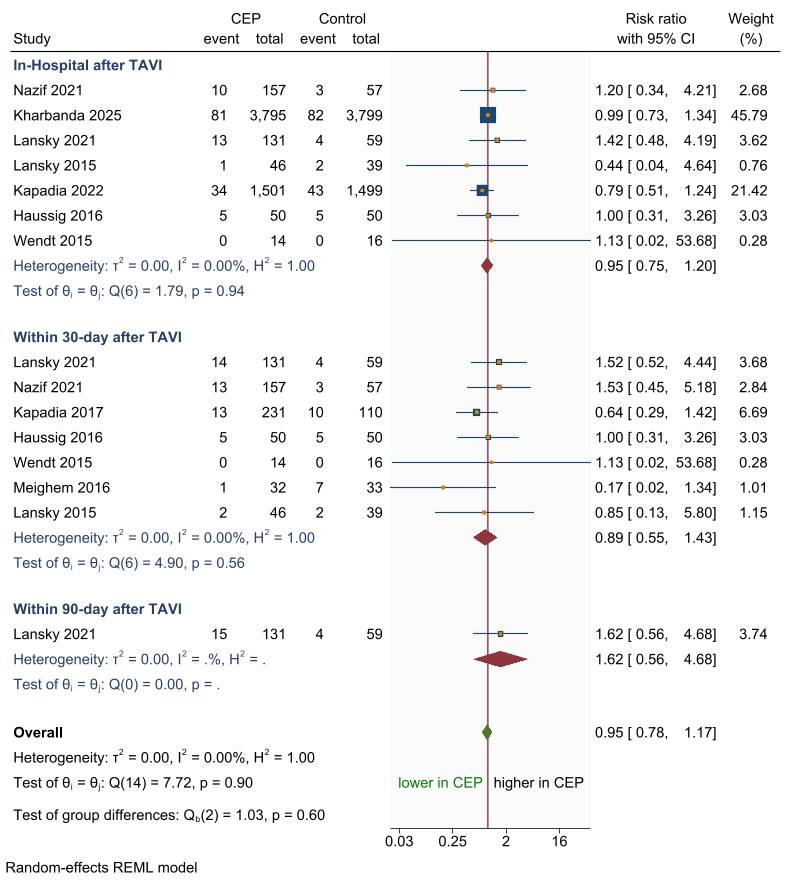


**Supplemental Figure 5:** Funnel plot for publication bias of all-cause stroke.


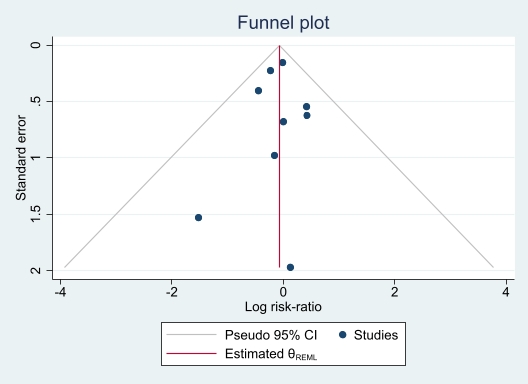


**Supplementary Figure 6:** Forest Plot meta-analysis for incidence of **(A)** cardiovascular mortality, **(B)** major adverse cardiovascular and cerebrovascular events (MACCE), **(C)** Major bleeding, and **(D)** Major vascular complications.


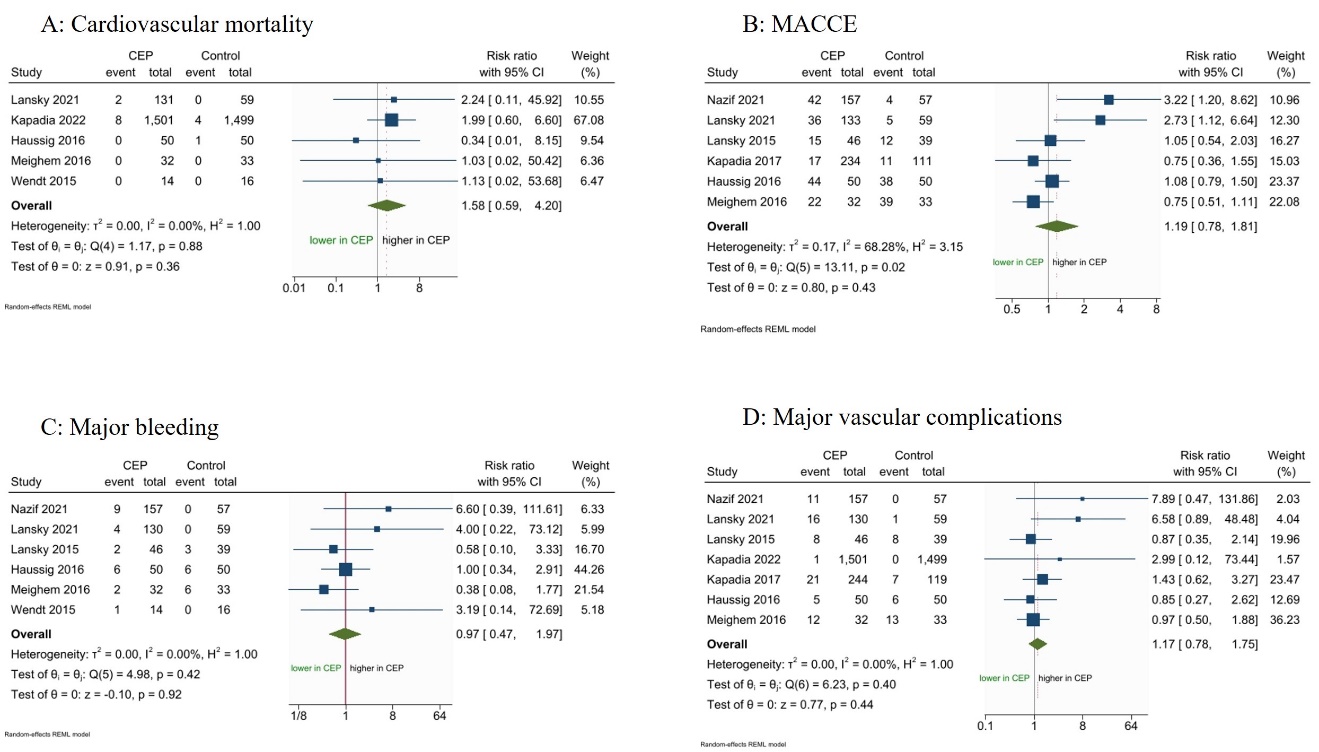

Supplement: Supplementary file 1 [file Datasheet1.docx]
